# Revisiting an Old Riddle: What Determines Genetic Diversity Levels within Species?

**DOI:** 10.1371/journal.pbio.1001388

**Published:** 2012-09-11

**Authors:** Ellen M. Leffler, Kevin Bullaughey, Daniel R. Matute, Wynn K. Meyer, Laure Ségurel, Aarti Venkat, Peter Andolfatto, Molly Przeworski

**Affiliations:** 1 Department of Human Genetics, University of Chicago, Chicago, Illinois, United States of America; 2 Department of Ecology and Evolution, University of Chicago, Chicago, Illinois, United States of America; 3 Howard Hughes Medical Institute, University of Chicago, Chicago, Illinois, United States of America; 4 Department of Ecology and Evolutionary Biology and the Lewis-Sigler Institute for Integrative Genomics, Princeton University, Princeton, New Jersey, United States of America

## Abstract

Understanding why some species have more genetic diversity than others is central to the study of ecology and evolution, and carries potentially important implications for conservation biology. Yet not only does this question remain unresolved, it has largely fallen into disregard. With the rapid decrease in sequencing costs, we argue that it is time to revive it.

## Abstract 2

With the recent revolution in sequencing, we revisit the unresolved question of what influences the range and values of genetic diversity across taxa.

What evolutionary forces maintain genetic diversity in natural populations? How do diversity levels relate to *census population sizes* (Box 1)? Do low levels of diversity limit adaptation to novel selective pressures? Efforts to address such questions spurred the rise of modern population genetics and contributed to the development of the *neutral theory of molecular evolution*—the null hypothesis for much of evolutionary genetics and comparative genomics [1]–[3]. Yet these questions remain wide open and, for close to two decades, have been neglected [4]. Most notably, little progress has been made to resolve a riddle first pointed out 40 years ago on the basis of *allozyme* data: the mysteriously narrow range of genetic diversity levels seen across taxa that vary markedly in their census population sizes [5]. This gap in our understanding is glaring, and may hamper efforts at conservation (e.g., [6]).

**Box 1. Glossary**

***Allozymes***: Allelic variants of a protein, often detected by differences in gel electrophoresis.

***Balancing selection***: Natural selection that maintains variation longer than expected from genetic drift alone.

***Census population size***: The actual number of individuals in a population; methods to estimate this number vary depending on the species and may involve aerial, transect, or capture/recapture counts.

***Diversity levels***: The measure used here is the probability that a pair of randomly chosen haplotypes differ at a site.

***Effective population size***: The size of an idealized population with some of the same properties as the actual one, e.g., the same rate of genetic drift. Under simplifying assumptions, notably a constant population size and no population structure, this parameter can be estimated from observed diversity levels, given an independent estimate of the mutation rate.

***Fluctuating selection***: When the fitness of an allele changes over time or over space.

***Genetic draft***: A dramatic loss of genetic variation due to strong, frequent selection at nearby sites [8].

***Genetic drift***: In a finite population, the loss of genetic variation due to the random sampling of gametes at each generation.

***Local adaptation***: Adaptation to a particular environment that is not shared by the entire species.

***Nearly neutral theory of molecular evolution***: A modification of the neutral theory, in which many mutations are slightly deleterious, rather than strictly neutral or strongly deleterious [75].

***Neutral theory of molecular evolution***: The theory that most genetic variation seen within populations and between species is neutral, and most mutations are either neutral or strongly deleterious [11].

***Panmixia***: Random mating among individuals, and hence no population structure.

***Phylogenetically independent contrasts***: A statistical method that allows one to compare properties of species controlling for their evolutionary relationship.

***Purifying (negative) selection***: Natural selection that favors the common, fitter allele against rare, deleterious alleles.

***Selection at linked sites***: Selection at sites linked to the locus under consideration, which can affect the population dynamics of alleles at that locus.

***Silent sites***: A general term for synonymous, intronic, and intergenic sites—all sites at which mutations do not change an amino acid.

***Variation-reducing selection***: Selection that leads to a decrease in diversity at linked sites.

With the recent technological revolution in sequencing, the data needed to address questions about the determinants of genetic diversity levels are now within reach. As a first step towards reviving these questions, we compile existing estimates of nuclear sequence diversity. These data are highly preliminary, but they underscore how little is known about the narrow span of diversity levels across species or why some species maintain more genetic variation than others [5],[7],[8], and they offer a glimpse of trends that may be worth pursuing.

## What We Expect from Simple Models

According to the neutral theory of molecular evolution, genetic diversity levels at neutral sites reflect a balance between mutational input and the loss of genetic variation due to the random sampling of gametes in a finite population (“*genetic drift*”) [9]–[11]. Under simplifying assumptions, the rate of genetic drift is inversely proportional to the population size. Equilibrium diversity levels are then given by the product of the constant census population size *N* and *u*, where *u* is the rate of mutation per generation. In reality, populations fluctuate in size over time and individuals can vary greatly in their reproductive success. Often, these and other deviations can be accommodated by substituting a much smaller “*effective population size*,” *N*_e_, for the census population size *N* [12]. A simple expectation is then that, all else being equal, species with larger and more stable census population sizes will tend to experience a smaller fluctuation in allele frequencies (i.e., larger *N_e_*), leading them to maintain greater levels of neutral genetic diversity.

At sites on which natural selection acts, however, the rate of loss of genetic variation will depend in more complex ways on the population size. Population genetic theory indicates that selection will be more effective in large, random-mating populations [13]. Whether this should result in a faster or slower rate of loss of genetic variation at selected sites is unclear, since some modes of selection (e.g., for a beneficial allele) lead to the loss of genetic variation, but others (e.g., *local adaptation* or *fluctuating selection*) can maintain it [13],[14].

These considerations matter in predicting diversity levels at sites directly under selection but also at nearby neutral sites, because selection at one site impacts variation at neighboring positions in the genome through linkage [7],[15],[16]. In a sense, selection at linked sites can be seen as an additional source of variance in reproductive success (i.e., as exacerbating drift) [17]. Thus, neutral diversity patterns will depend not only on the rate of genetic drift, but also on the rate at which variation is lost due to *selection at linked sites*; that is, they will depend on the frequency and strength of selection and the distribution of selected loci throughout the genome [17]. Even if the proportion of sites under direct selection is relatively small, the impact on genome-wide diversity may be substantial. Which modes of natural selection predominate will also be important: for instance, neutral diversity levels will be more greatly reduced if adaptation acts on new mutations rather than standing variation (e.g., [18]), and may be increased if *balancing selection* is common [12],[14]. The influence of all these factors remains largely unknown, even in the best-studied organisms.

## An Emerging Role for Natural Selection

What has become clear over the past decade or so is that signatures of natural selection are widespread: in *Drosophila* species, notably, the analysis of polymorphism and divergence suggests that half of the amino acid substitutions between species have been fixed by selection (recently reviewed in [19],[20]). This fraction varies markedly across taxa, with similar estimates seen in wild mice, *Capsella grandiflora*, and *Escherichia coli* [19],[21],[22] but dropping to 0%–10% in humans (e.g., [23],[24]), yeast (e.g., [25]), and a variety of plant species [26]. Analyses of diversity patterns along the genome also point to pervasive selective effects. Notably, diversity (but not divergence between species) is lower in regions of low recombination in many taxa, including *Drosophila* species ([27],[28]; recently reviewed in [20]), humans [29], *Caenorhabditis elegans* [30], and sparrows [31], with a much weaker pattern seen in yeast [32], and no detectable relationship in wild species of tomato (when corrected for divergence levels) [33], *Arabidopsis lyrata* [34], or *A. thaliana* [35]. The most likely explanation for the reduced diversity in regions of low recombination is that a given neutral site is linked to more selected alleles. Thus, these observations support the prevalence of a form of selection that reduces variation at linked neutral sites [16],[27],[28] and point to intriguing differences among taxa.

While evidence for the prevalence of *variation-reducing selection* is mounting, there is still no consensus about what form predominates: in particular, whether patterns of variation are shaped primarily by *purifying selection* or by some mode of positive selection (reviewed in [20],[36]). If positive selection at linked sites is the main form, then differences in diversity among taxa could be due to higher rates of adaptation in outbred species with larger census population sizes or weaker population structure (e.g., widespread dispersal of gametes) [19],[37],[38]. Species may also differ in their dominant modes of selection, depending on ecology, life history, or genetic constraints (e.g., [39]–[42]). As one example, species with larger population sizes may have more standing variation with which to respond to novel selection pressures, leading to a smaller fraction of adaptations from new mutations [40]. In turn, species with larger geographic ranges may be more likely to adapt through multiple, geographically restricted mutations than by global sweeps [42]. In both cases, adaptation may have less of an effect in reducing variation than expected under assumptions of *panmixia* and selection on new mutations.

## The Little We Know about Genetic Diversity across Species

Before a general theory of the ecological and genetic determinants of diversity levels can be constructed, we need a systematic survey of diversity across a wide range of taxa. Such a survey was a central agenda for two decades of molecular population genetics (see Box 2). The most recent technological revolution in sequencing enables these questions to be revisited on an unprecedented scale, using nucleotide variation data. To motivate such data collection, we built a comprehensive compilation of available estimates of nuclear *diversity levels* in eukaryotes, treating autosomes and sex chromosomes separately (and excluding data from heterogametic sex chromosomes, Dataset S1; see Text S1 for our criteria and for a list of smaller data sets of this kind). In order to obtain less noisy estimates, we only considered surveys of three or more nuclear loci, and to consider variation on sites that are likely to be under less direct selection, we focused on estimates for *silent sites* (when possible, on synonymous sites; see Text S1 and Table S1 for a comparison of estimates from different types of sites). In this regard, there may be no ideal choice of annotation, as even synonymous sites are constrained by codon bias and other selective pressures (reviewed in [43]). Nonetheless, this compilation should provide a rough sense of how much neutral diversity levels vary across available taxa, and so, these caveats notwithstanding, we used these estimates as measures of “neutral diversity.”

**Box 2. Allozyme Studies and Their Limitations**

Starting with the introduction of methods to characterize protein variation in 1966, allozymes were used to estimate genetic diversity levels in hundreds of species, evaluate trends, and compare observations to predictions of simple population genetic models [5],[83]. At the time, the high levels of variation prompted debate between models in which selection directly maintains variation (the “balance school”) and the neutral theory, in which selective effects are negligible [4],[5],[11],[84]. In addition, the range of diversity levels across taxa was surprisingly small—much too small to reflect the span of census population sizes in any simple way. Much speculation followed about the extent to which diversity levels were correlated with census population size or other demographic and ecological factors (e.g., [49]). But allozyme data have a number of limitations; in particular, not all genetic changes are detectable [85]. Moreover, allozyme variation may often not be neutral [86], making it difficult to disentangle the effects of direct selection on protein variation and selection at linked sites ([14], Chapter 1.3). Because of these technical limitations and the rise of the neutral theory, by the mid-80s, efforts to understand the determinants of diversity were waning, and the questions left open [4],[84].

**Figure 1:**
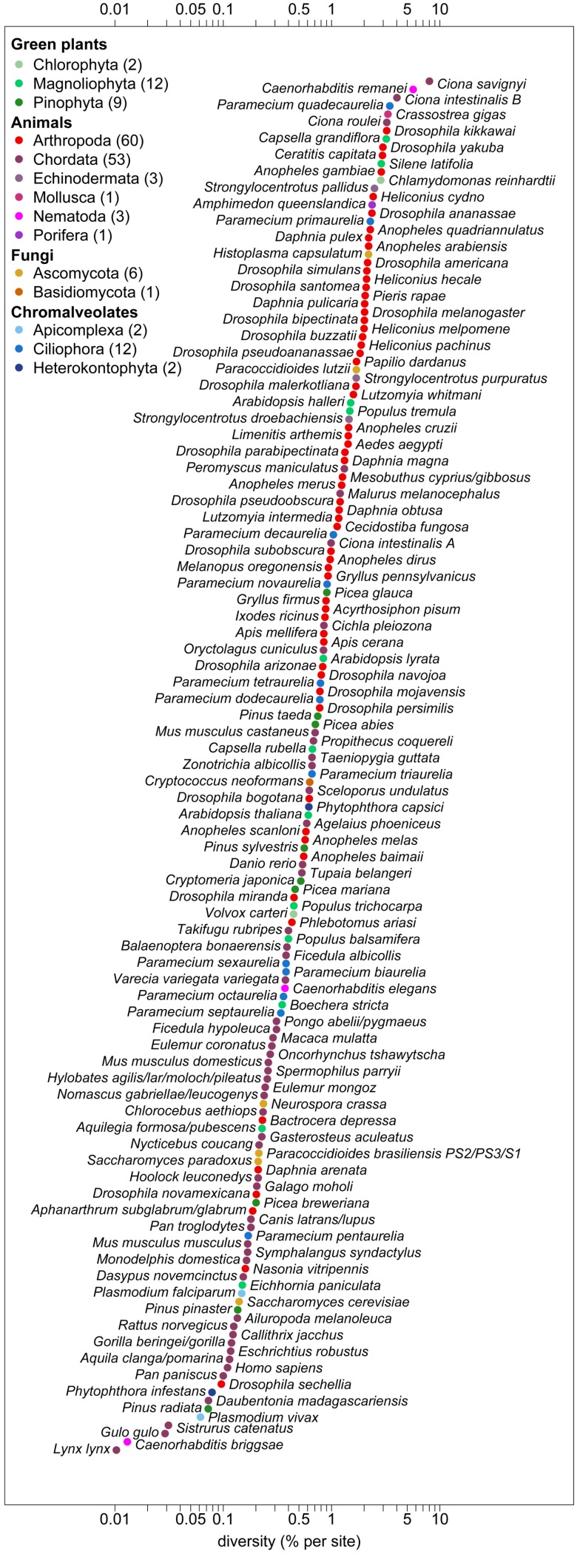
Autosomal nucleotide diversity levels across species. Autosomal genetic diversity is given as the average number of pairwise differences per base pair, in percent, and is shown on a log10 scale. Each estimate represents the mean of at least three loci and in most cases is based on only non-coding or synonymous sites. The estimates are ordered by diversity level, labeled by species name, and colored by the phylum to which each species belongs. The number of species in each phylum is given in parentheses in the legend.

In total, we were able to compile autosomal estimates for 167 species distributed in 14 phyla, including whole genome diversity data for a dozen species but very limited data for taxa other than *Drosophila* and mammals (see Figure 1). We focused on within-population diversity levels, which should be less sensitive to migration rates than estimates from pooled population samples ([12]; see Text S1 and Table S2). We then used these estimates to examine the relationships between neutral genetic diversity and several ecological parameters that may be associated with differences in census population sizes or other influential factors such as population structure.

**Figure 2:**
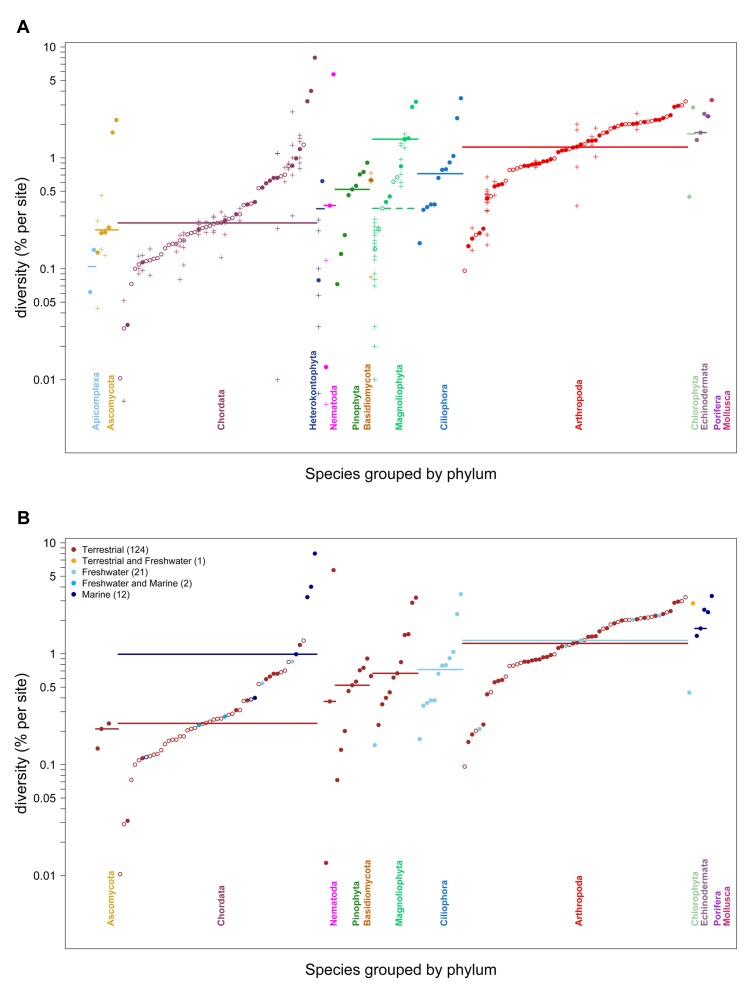
Autosomal nucleotide diversity levels across species, grouped by phylum. Diversity estimates for each species are the same as in Figure 1; here they are ordered within phylum, and phyla are presented in order of their median diversity levels. Within Chordata, open circles indicate mammals, and within Arthropoda, they denote *Drosophila* species. We note that the three most diverse chordates are all invertebrate sea squirts. In panel (A), estimates are colored by the phylum to which each species belongs and horizontal bars mark the median estimate for each phylum; for Magnoliophyta, a dashed line marks the median for selfing species (open circles) and a solid line marks the median for outcrossing species. (We do not provide *p* values for comparisons because of the lack of phylogenetic independence.) Crosses denote estimates for individual populations and are shown when population structure was reported in the original study. In panel (B), estimates are colored according to whether the species lives in a terrestrial, freshwater, or marine environment (not all species are categorized). Horizontal bars indicate the median for each category within each phylum (only shown when more than two species fall in the category). The number of species in each habitat is given in parentheses in the legend.

In spite of the spotty nature of the data, some broad patterns are consistent with the notion that species with larger census sizes harbor more neutral genetic diversity. Across phyla, arthropods tend to have higher nucleotide diversity (with a median of 1.25% per base pair) than do chordates (0.26%), and plants fall in the middle (1.48% for outcrossing Magnoliophyta and 0.52% for Pinophyta) (Figure 2A; see Figure S1 for sex chromosomes). The same ordering was seen with allozyme data (but not mtDNA) [44],[45]. In fact, across 22 species for which both estimates exist, allozyme and nucleotide estimates are correlated (Spearman's ρ = 0.33, one-tailed *p* = 0.068; Figure S2A, see also [45]; for mtDNA, see [46]–[48]), with slightly more variation across species seen in nucleotide than allozyme data (Figure S2B).

Within a single phylum, there is a broad range of nucleotide diversity levels, with almost the same span as seen across phyla (e.g., comparing Nematoda and Magnoliophyta; [44]). This observation indicates that, whatever the determinants of genetic diversity levels, they vary among species within a phylum as well as among phyla. Practically, it suggests that future studies contrasting diversity patterns among closely related species should be informative about influential factors.

## Ecological and Life-History Correlates of Genetic Diversity

At the level of analysis afforded here (i.e., without *phylogenetically independent contrasts*), a few intriguing observations emerge: the species in the four most diverse phyla live mainly in marine or freshwater environments (Figure 2B), as observed with allozymes [49],[50], and the marine and freshwater species are on average more diverse than the terrestrial species within Chordata (and barely, within Arthropoda) (Figure 2B).

**Figure 3:**
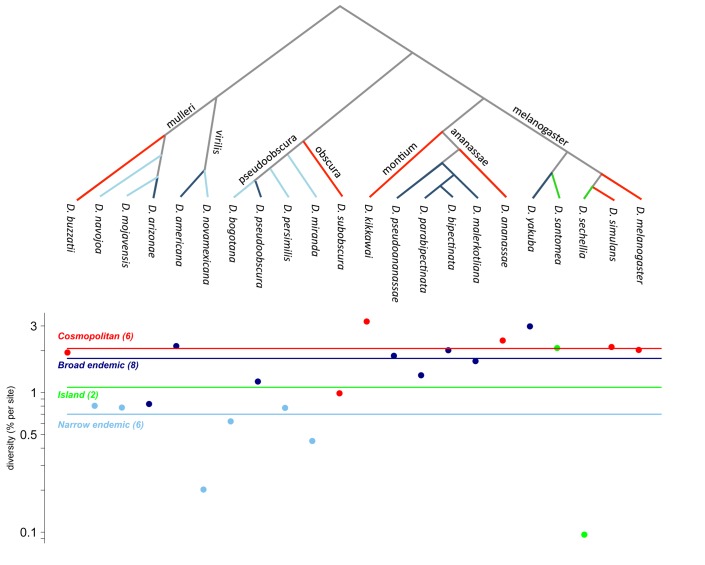
*Drosophila* species phylogeny (top), autosomal nucleotide diversity estimates (bottom), and geographic ranges. Diversity levels are significantly correlated with the range category a priori ordered as island, narrow endemic, broad endemic, cosmopolitan using a generalized least squares method, and controlling for the phylogeny (*F* = 21.49, *df* = 20, *p* = 0.0002). Names along the branches of the phylogeny identify the *Drosophila* subgroup to which the species below belong(s); branch lengths displayed are arbitrary. For a definition of the four range categories, see Text S1. Horizontal lines mark the median diversity of species within each range category. We note that the estimates for *Drosophila buzzatii* and *Drosophila subobscura* include loci within polymorphic inversions and represent the average diversity within a chromosomal arrangement.

The geographic range of a species also appears to be influential [44],[51]. Specifically, within *Drosophila*, where range categories are well distributed across the phylogeny, cosmopolitan species are more diverse than broad endemics, which are in turn more diverse than narrow endemics (Figure 3; *F* = 21.49, *p* = 0.0002 using a phylogenetic generalized least squares approach with 20 *df*; see Text S1). Since a number of *Drosophila* species have expanded their ranges relatively recently [52] and therefore are unlikely to be at mutation-drift equilibrium, the observed pattern could result from the sizes of the ancestral populations.

In addition to these ecological factors, life history traits such as mating system are expected to have a discernable effect on genetic diversity [53]. Notably, self-fertilization is expected to affect neutral diversity because inbreeding reduces *N_e_*—under complete inbreeding to half its value [54]. If selection that reduces variation at linked sites is widespread, the lower effective recombination in self-fertilizing species could also reduce neutral diversity by accentuating the effects of selection on linked sites [12]. In accordance with these predictions, among the 12 species of flowering plants in our datase, selfers have lower diversity (the median is 0.35% per bp) than obligate outcrossers (1.48%) (see Figure 2A and Figure S3 for more detail; e.g., [55],[56]). Moreover, the difference in diversity between closely related species that differ in mating system is in some cases greater than 2-fold (e.g., *Capsella rubella* versus *C. grandiflora*; [57]). Observations from flowering plants are therefore consistent with a role for selection at linked sites [12]. Alternatively, ecological explanations might also account for the extreme effects of selfing: for example, selfers might experience greater fluctuation in population size due to more frequent extinction/recolonization events [53].

**Figure 4:**
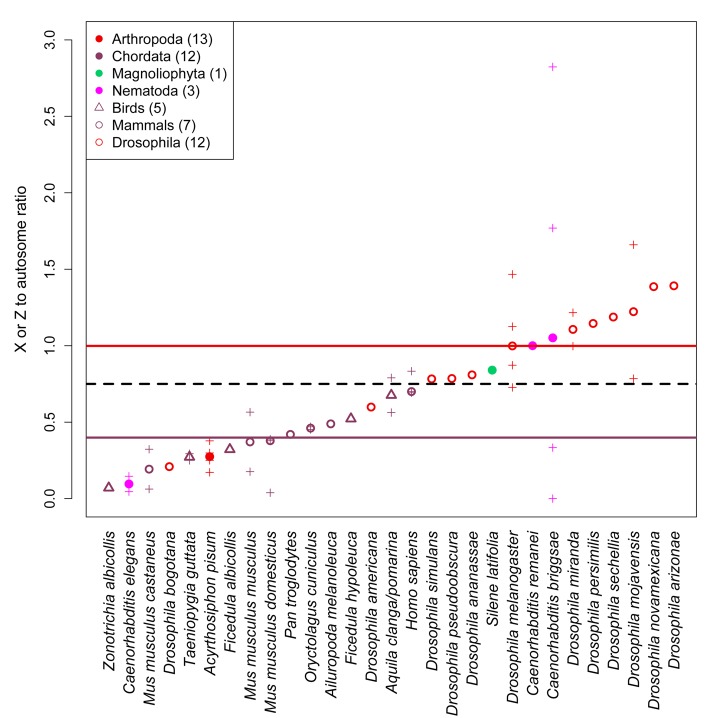
Comparison of autosome and sex chromosome nucleotide diversity. The ratio of sex chromosome to autosome diversity is plotted for the 29 species in which both estimates were available from the same population(s). Colors indicate the phylum to which the species belong. Within Chordata, open circles denote mammals and open triangles birds; within Arthropoda, open circles denote *Drosophila* species. The number of species in each group is given in parentheses in the legend. Within species, crosses represent the ratio estimated from different populations, with the median of the estimates shown as a triangle (birds) or circle (all other species). Solid horizontal lines indicate the median sex chromosome to autosome ratio for arthropods and chordates, colored as in the key. The black dashed line indicates where sex chromosome diversity equals three-fourths of autosomal diversity.

Finally, across taxa with heterogametic sexes, sex chromosomes show different patterns relative to autosomes (Figure 4). Making a number of simplifying assumptions—in particular, no natural or sexual selection and no differences in mutation rates between sexes—the different numbers of sex (X or Z here) chromosomes versus autosomes in the population predict a ratio of sex chromosome to autosome diversities of 3∶4 (reviewed in [12],[58]). Though our estimates of this ratio are noisy, in *Drosophila*, the ratio tends to be close to 3∶4 or higher (the mean is 0.97; by a sign test for a difference from 0.75, the two-tailed *p* = 0.039). This pattern might reflect a larger variance in male reproductive success relative to females or other demographic effects [12]. In mammals, the mean is lower (0.43; two-tailed sign test *p* = 0.016), in principle consistent with a ratio of 3∶4 and a (much) lower mutation rates in females, where the X is found two-thirds of the time [59]. However, the ratio of diversity on the sex chromosome (Z) relative to the autosomes also appears to be lower than 3∶4 in birds (the mean is 0.37; combining our five data points with the six more recently collected by [60], the two-tailed sign test *p* = 0.001), even though the Z chromosome spends most of its time in males, who are thought to have a higher mutation rate [61]. High variance in male reproductive success could contribute to a lower ratio in these birds, but the most extreme case would only drive the ratio to 9∶16 [12], and the male-biased mutation rate inferred from substitution rates would raise this lower limit. Thus, it appears that existing patterns may be difficult to explain by sex differences in mutation rates or offspring numbers alone, supporting prevalent effects of variation-reducing natural selection on sex chromosomes (but see [60]).

## The Enduring Riddle

In addition to these crude patterns, the compilation of silent diversity levels makes evident the same puzzle as seen in the allozyme data [5]: the range of neutral diversity levels across taxa is much smaller than expected from the huge variation in current census population sizes. Among the 167 species that met our criteria for the autosomes, nucleotide diversity π ranges from 0.01% per base pair in *Lynx lynx* to 8.01% in *Ciona savignyi*, a span of only 800-fold (Figure 1). While census population sizes for entire species are difficult to measure and even the few available estimates (mainly birds and mammals) may be unreliable, an 800-fold range is likely many orders of magnitude smaller than expected. As an illustration, diversity levels in the gibbon *Hoolock leuconedys* are 0.21% for a current census population size estimate of 10,000–50,000 individuals (http://www.iucnredlist.org), whereas in *Drosophila buzzatii*, a species distributed worldwide, they are only ∼10 times higher (1.94%) when population size estimates are on the same order *per hectare* [62]. While some of the difference could be due to a recent decline in gibbons or increase in *D. buzzatii*, the census population sizes are unlikely to have ever been within an order of magnitude.

A possible explanation for the narrow range of diversity levels is that nuclear mutation rates per generation vary inversely with effective population size (e.g., due to more effective selection for a lower mutation rate in species with higher effective population sizes) [63],[64]. Direct estimates are limited, but suggest that the nuclear mutation rate per generation ranges over 100-fold, from 3.3×10^−10^ per site in *Saccharomyces cerevisiae* to 3.5×10^−9^ in *Drosophila melanogaster*, 1.3×10^−8^ in *Homo sapiens*, and 3.8×10^−8^ in *Mus musculus* [63],[65], potentially consistent with this explanation (with the caveat that the per generation mutation rate may not be the relevant time-scale for *S. cerevisiae*). If across taxa there is a systematic relationship between mutation rate and population size, it would lead large populations to have relatively low diversity levels and thus to a smaller range of diversity levels than population sizes [63]. Although it may be important, this explanation seems unlikely to entirely resolve the riddle. For instance, the flycatcher *Ficedula albicollis* is estimated to have an approximate population size of four to seven million and the sparrow *Zonotrichia albicollis* of 140 million (http://www.birdlife.org). Mutation rates in these two bird species are unknown but presumably similar, yet average diversity levels differ by less than 2-fold (0.38% versus 0.66% per bp, respectively). As another illustration, within *Drosophila*, there is a surprisingly small (although significant) difference between the extremes of narrow endemics and cosmopolitan species (3-fold; see Figure 3), which presumably have vastly different census population sizes.

## Possible Resolutions of the Riddle

Why then are neutral diversity levels and allozyme variation contained within such a narrow range? If neutral diversity levels are indicative of the ability of a species to adapt to novel selective pressures, then, as argued in the context of conservation biology, there may be a lower limit beyond which a species cannot maintain the variation necessary to respond to a change in environment and so is rapidly driven to extinction (e.g., [6]). In turn, there may be upper limits imposed by functional or structural constraints; for example, excessive heterozygosity could interrupt chromosome pairing [66] or lead to reproductive incompatibilities between individuals living in distant regions of the species' range (e.g., [67]). Another explanation for the upper limit could be that effective population sizes increase extremely slowly with the census population sizes, for example if species that are more numerous experience more frequent or more extreme population bottlenecks, and so remain further from their mutation-drift equilibrium diversity levels [11],[68].

Alternatively, the narrow range of diversity may be due to the effects of selection at linked sites. That habitat and range are predictive of diversity is consistent with a neutralist scenario in which aquatic species, species with larger ranges, or outcrossers have greater and more stable population sizes and therefore maintain higher neutral diversity, but it may also be consistent with models in which positive selection is ubiquitous. Under certain assumptions, widespread adaptation can constrain the range of neutral diversity across species: when adaptation is limited by the input of new mutations, larger populations experience a greater influx of beneficial mutations and therefore greater effects of variation-reducing selection (“*genetic draft*”) at linked neutral sites [8]. In other words, under certain assumptions, there is more genetic draft in species that experience less genetic drift, and combined, these two evolutionary forces lead to a smaller range of neutral diversity across species than expected from differences in their census population sizes [8]. Higher diversity might then be observed in species with broader ranges because local adaptation maintains variation, or because global selection (and the associated loss of diversity at linked sites) is hindered by population structure [42]. As summarized above, several lines of evidence are consistent with marked effects of selection on diversity levels. Nonetheless, the genetic draft explanation requires strong, frequent selection that reduces diversity levels by orders of magnitude, when the few available estimates (based on contrasting diversity levels in different genomic backgrounds) suggest a much weaker impact [69]–[72]. Thus, it remains unclear whether plausible selection models can readily explain the narrow range of diversity among species.

Selection on silent sites themselves may also be a factor contributing to the narrow range of diversity across species. It is well established that codon bias and other selective pressures constrain the evolution of synonymous sites, and that many sites in non-coding regions are subject to purifying selection (e.g., [43],[73],[74]). If a subset of the mutations at silent sites is strongly deleterious in all species, diversity levels would be decreased relative to strict neutrality, but nonetheless they would increase linearly with the effective population size [11]. If, however, a substantial fraction of silent sites are weakly selected (with |2*N*_e_s|<1) and therefore under more effective purifying selection in larger populations, diversity levels may increase much more slowly with *N*_e_ [13],[75]. While a *nearly neutral model* could in principle help to account for a reduced range of diversity levels, such an explanation raises the question already voiced by Gillespie and Ohta 15 years ago: “Why should nature conspire to have the value of 2*N*_e_s fall within such a narrow window for most creatures?” [76].

## Where to from Here?

The central puzzle remains: both allozyme and diversity levels at sites less likely to be directly affected by selection vary surprisingly little among species, and mostly in ways that we still do not understand. This puzzle has persisted for close to half a century because it is a difficult one, and simply gathering more data will not resolve it. However, characterizing diversity levels along the genomes of thousands of species is a necessary first step, and now a feasible one. In fact, data collection is already on its way, with hundreds of genome sequences now available, and the proposal to scale up to 10,000 species, sampled throughout the plant and animal kingdoms [77]. This effort will provide a necessary scaffold on which to build comparative population genomics, but it will need to be complemented by numerous population surveys, with careful geographic sampling. It will also have to be accompanied by the study of closely related species that differ in potentially relevant ecological or life history traits or in genome architecture (e.g., [78]).

In addition to enabling population-level sequence data, the revolution in sequencing will also permit the estimation of de novo mutation rates (as done, e.g., in humans [79],[80]). Knowledge of the mutation rate across many species will allow diversity levels to be compared to census population sizes without the confounding effect of differences in mutation rates. With better genome annotations (including genetic maps), it may also become possible to identify sites not closely linked to any functional elements, providing an estimate of neutral diversity unaffected by selection. The plausibility of the genetic draft hypothesis can then be evaluated by quantifying the effects of selection in regions of the genome more or less sheltered from the effects of natural selection, for example sites at varying genetic distance from functional elements.

With genome-wide polymorphism data and mutation rate estimates from many species, hypotheses about the ecological and genetic determinants of diversity levels will become testable. As one example, the major features of the demographic history of species can be inferred (e.g., [81]) and integrated with independent reconstructions of ancestral ranges (e.g., [82]) in order to assess whether species with larger census sizes are less stable. In addition to these analyses, new theory will be needed to relate ecological and life history factors to modes of selection and the patterns of genetic variation seen across organisms. Such studies may not provide a universal answer, but regardless they will help fill a gaping hole in our understanding of genetic variation and its determinants.

## Supporting Information

Dataset S1 Nucleotide diversity estimates.
(TXT)

Figure S1 Nucleotide diversity estimates for sex chromosomes (X or Z) across species. Each estimate represents the mean of at least three loci on the X or Z chromosome and is based on silent sites or the entire chromosome in all but four cases. The estimates are colored by the phylum to which each species belongs and within phylum are ordered by diversity level; phyla are ordered by their median diversity level, shown as a horizontal bar. Crosses indicate estimates for individual populations when population structure was reported in the original study. Within Chordata, open circles denote mammals and triangles birds; within Arthropoda, open circles denote *Drosophila*. The estimate of 0 for *Drosophila sulfurigaster bilimbata* (based on five loci) is not shown.
(TIF)

Figure S2 Comparison of nucleotide diversity and allozyme heterozygosity. Autosomal nucleotide diversity estimates are from the current compilation and allozyme heterozygosity estimates are from [44]; only the 22 species in both studies are included. In panel (A), the nucleotide diversity and allozyme heterozygosity estimates are plotted for each species (Spearman's ρ = 0.33, one-tailed *p* = 0.068). Open circles represent *Drosophila* (within Arthropoda) and mammals (within Chordata). In panel (B), the distribution of nucleotide diversity (left) and allozyme heterozygosity (right) across species are shown, with the medians represented at the same level as a black bar. The number given at the bottom is the coefficient of variation.
(TIF)

Figure S3 Autosomal nucleotide diversity by mating system in flowering plant species. Genetic diversity estimates for species in the phylum Magnoliophyta, colored according to whether the mating system allows for self-fertilization. Horizontal lines indicate the median genetic diversity for each of the two categories.
(TIF)

Table S1 The median nucleotide diversity within a phylum considering estimates based on all site types versus only synonymous sites. Listed are phyla in which at least three species have a synonymous diversity estimate and estimates for multiple types of sites are represented.
(DOC)

Table S2 The median nucleotide diversity within a phylum considering estimates based on sampling a single population versus sampling multiple populations with no observed population structure. Listed are phyla with at least two species in each group.
(DOC)

Text S1 Supporting information and methods.
(DOC)
