# Supplementary figures and images for: Revisiting an Old Riddle: What Determines Genetic Diversity Levels within Species?
Source: PLoS Biol. 2012 Sep 11;10(9):e1001388. doi: 10.1371/journal.pbio.1001388 (PMC3439417; doi:10.1371/journal.pbio.1001388)

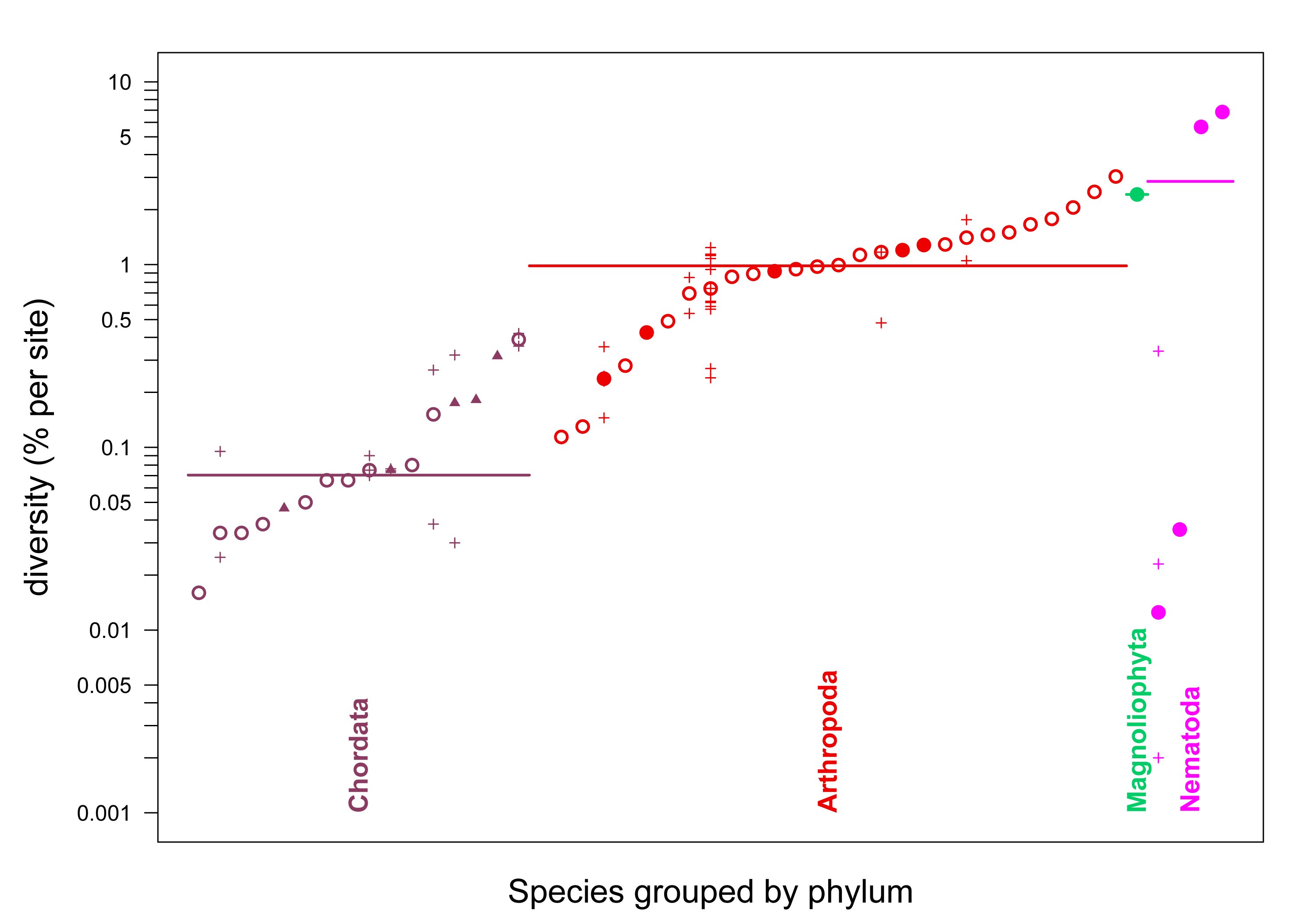

Supplement: Figure S1 — Nucleotide diversity estimates for sex chromosomes (X or Z) across species. Each estimate represents the mean of at least three loci on the X or Z chromosome and is based on silent sites or the entire chromosome in all but four cases. The estimates are colored by the phylum to which each species belongs and within phylum are ordered by diversity level; phyla are ordered by their median diversity level, shown as a horizontal bar. Crosses indicate estimates for individual populations when population structure was reported in the original study. Within Chordata, open circles denote mammals and triangles birds; within Arthropoda, open circles denote Drosophila. The estimate of 0 for Drosophila sulfurigaster bilimbata (based on five loci) is not shown. (TIF) [file pbio.1001388.s002.tif]

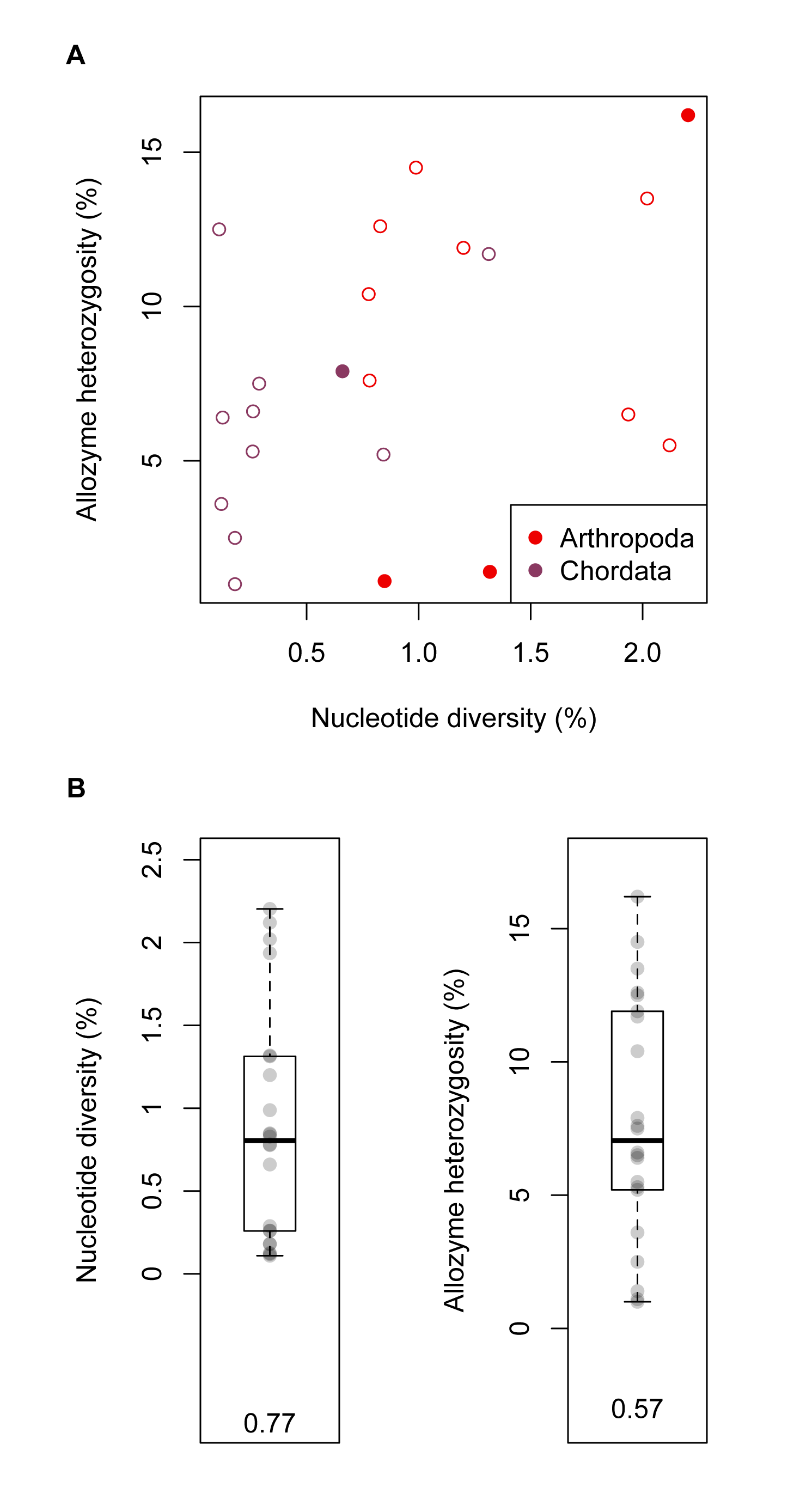

Supplement: Figure S2 — Comparison of nucleotide diversity and allozyme heterozygosity. Autosomal nucleotide diversity estimates are from the current compilation and allozyme heterozygosity estimates are from [44]; only the 22 species in both studies are included. In panel (A), the nucleotide diversity and allozyme heterozygosity estimates are plotted for each species (Spearman's ρ = 0.33, one-tailed p = 0.068). Open circles represent Drosophila (within Arthropoda) and mammals (within Chordata). In panel (B), the distribution of nucleotide diversity (left) and allozyme heterozygosity (right) across species are shown, with the medians represented at the same level as a black bar. The number given at the bottom is the coefficient of variation. (TIF) [file pbio.1001388.s003.tif]

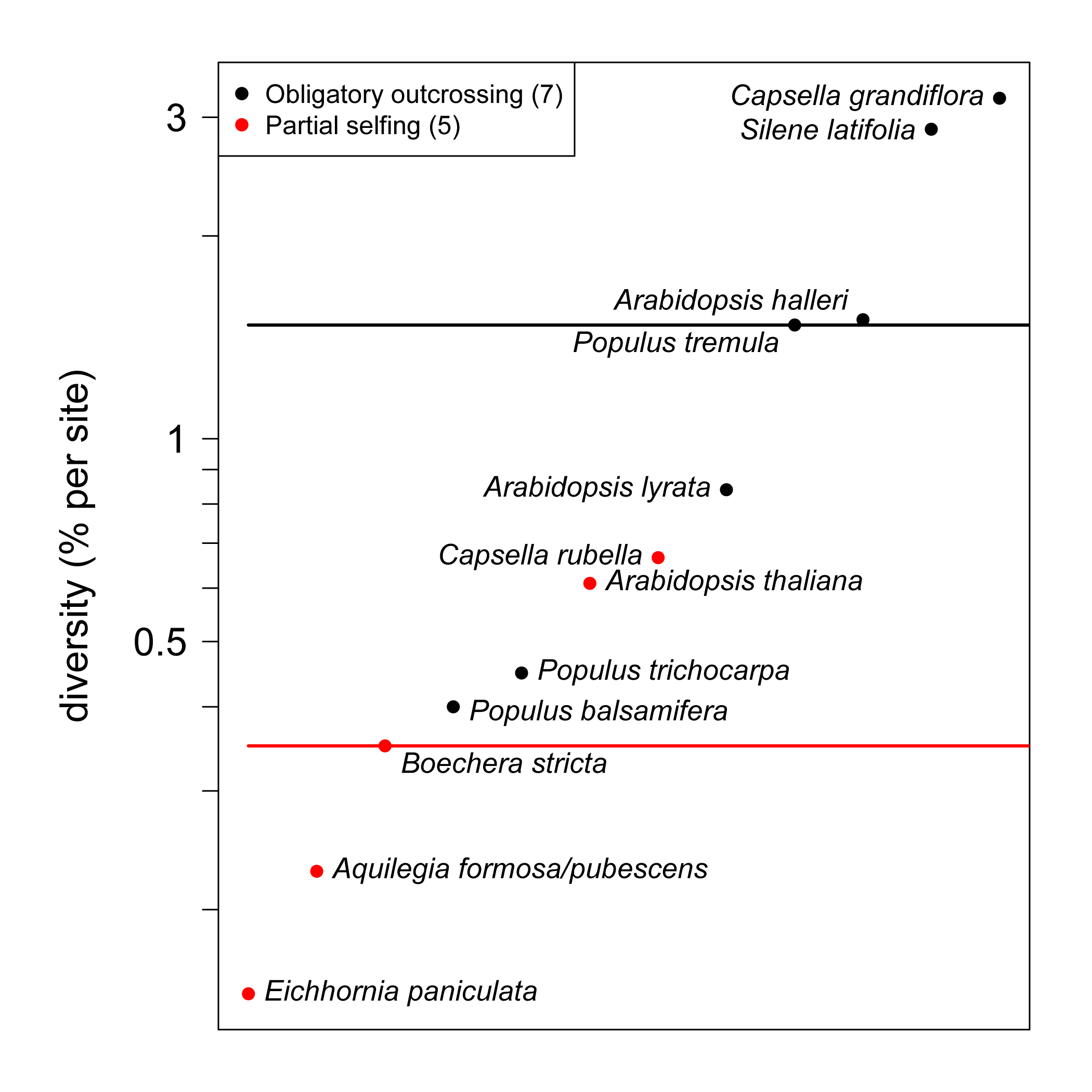

Supplement: Figure S3 — Autosomal nucleotide diversity by mating system in flowering plant species. Genetic diversity estimates for species in the phylum Magnoliophyta, colored according to whether the mating system allows for self-fertilization. Horizontal lines indicate the median genetic diversity for each of the two categories. (TIF) [file pbio.1001388.s004.tif]
