# Supplementary material for: Revisiting an Old Riddle: What Determines Genetic Diversity Levels within Species?
Source: PLoS Biol. 2012 Sep 11;10(9):e1001388. doi: 10.1371/journal.pbio.1001388 (PMC3439417; doi:10.1371/journal.pbio.1001388)
Supplement: Table S1 — The median nucleotide diversity within a phylum considering estimates based on all site types versus only synonymous sites. Listed are phyla in which at least three species have a synonymous diversity estimate and estimates for multiple types of sites are represented. (DOC) [file pbio.1001388.s005.doc]

| Phylum | # species with a diversity estimate based on | | Median diversity (%) considering estimates based on | |
| --- | --- | --- | --- | --- |
|  | Any type of site | Only synonymous sites | Any type of site | Only synonymous sites |
| Arthropoda | 60 | 13 | 1.25 | 2.04 |
| Chordata | 53 | 19 | 0.26 | 0.24 |
| Magnoliophyta | 12 | 5 | 0.64 | 0.55 |
| Pinophyta | 9 | 4 | 0.52 | 0.25 |
