# Supplementary material for: Revisiting an Old Riddle: What Determines Genetic Diversity Levels within Species?
Source: PLoS Biol. 2012 Sep 11;10(9):e1001388. doi: 10.1371/journal.pbio.1001388 (PMC3439417; doi:10.1371/journal.pbio.1001388)
Supplement: Table S2 — The median nucleotide diversity within a phylum considering estimates based on sampling a single population versus sampling multiple populations with no observed population structure. Listed are phyla with at least two species in each group. (DOC) [file pbio.1001388.s006.doc]

| Phylum | # species with a diversity estimate based on | | Median diversity (%) of species with estimates based on | |
| --- | --- | --- | --- | --- |
|  | A single population | Multiple populations and no population structure | A single population | Multiple populations and no population structure |
| Arthropoda | 9 | 40 | 1.20 | 1.32 |
| Chordata | 12 | 10 | 0.29 | 0.61 |
| Pinophyta | 2 | 5 | 0.32 | 0.52 |
