# Supplementary material for: Revisiting an Old Riddle: What Determines Genetic Diversity Levels within Species?
Source: PLoS Biol. 2012 Sep 11;10(9):e1001388. doi: 10.1371/journal.pbio.1001388 (PMC3439417; doi:10.1371/journal.pbio.1001388)
Supplement: Text S1 — Supporting information and methods. (DOC) [file pbio.1001388.s007.doc]

**Supporting Information** for

Revisiting an old riddle: what determines genetic diversity levels within species?

Ellen M. Leffler^1*^, Kevin Bullaughey^2+^, Daniel R. Matute^1+^, Wynn K. Meyer^1+^, Laure Ségurel^1,3+^, Aarti Venkat^1+^, Peter Andolfatto^4^ and Molly Przeworski^1,2,3*^

^1^ Department of Human Genetics, University of Chicago, Chicago, IL 60637, USA

^2^ Department of Ecology and Evolution, University of Chicago, Chicago, IL 60637, USA

^3^ Howard Hughes Medical Institute, University of Chicago, Chicago, IL 60637, USA

^4^ Department of Ecology and Evolutionary Biology and the Lewis-Sigler Institute for Integrative Genomics, Princeton University, Princeton, NJ 08544, USA

^+^ Contributed equally

^*^ To whom correspondence should be addressed:

Emails: [emleffler@uchicago.edu](mailto:emleffler@uchicago.edu), [mfp@uchicago.edu](mailto:mfp@uchicago.edu)

Data Collection

We searched for journal articles (through June 2011) that presented nucleotide diversity estimates of eukaryotic species, excluding species that had been domesticated (because of artificial selection and possible introgression from wild progenitors). We did not consider prokaryotes in our dataset because, in addition to the problem of defining species, they have a number of properties that likely make their population dynamics quite distinct from those of most eukaryotes (including huge population sizes, high mutation rates and tight linkage) [1,2] (see [3] for some estimates in prokaryotes). We kept only those cases where estimates were based on three or more nuclear loci that were not known to be closely linked. Where possible, we used estimates of π, the average number of pairwise differences [4], rather than θ_w_ [5], to diminish the effect of sample size. We required that the data be obtained by either DNA or RNA sequencing, thus excluding any estimates based on single nucleotide polymorphism genotyping, microsatellites, or other repeat typing. In order to minimize the effects of selection on the sites themselves, we did not consider estimates for non-synonymous sites or known untranslated regions; however, in a relatively small number of cases in which no other estimates were available, we included estimates for a mixture of non-synonymous and silent sites (type “mixed”, 40 estimates) or loci for which the annotation was unknown or not provided (type “anonymous” or “unclear”, 12 estimates). All other estimates were based on synonymous, intronic, intergenic or silent (a combination of the previous) sites or for the whole genome. In species with sex chromosomes, autosomes and sex chromosomes (X and Z) were treated separately. We did not collect estimates for chromosomes with little or no crossing-over (mtDNA, Y, W, or *Drosophila* fourth and neo-X chromosomes), since they are also expected to have different population dynamics (recently reviewed in [6]). The data are available in Dataset S1.

Species estimates

In order to obtain a diversity estimate for each species, we proceeded as follows. First, for each type of site in each study, we took the mean π (unweighted, where provided) over the loci sequenced except (1) when sets of loci were chosen because of the local recombination rate, in which case we only included those in regions of non-reduced recombination (to minimize the effects of linked selection and make the loci more comparable across species) and (2) when only mixed estimates were provided and at least three loci were known to contain <5% non-synonymous sites, in which case we took the mean of only those loci with <5% non-synonymous sites. For species with estimates for multiple types of sites, we took the median of estimates for non-overlapping types.

Often, multiple populations were sampled in a single study. If there was no population structure detected, we took the diversity estimate reported for the combined populations. When population structure was detected and separate estimates were available, we recorded each estimate as well as the median across populations. Finally, when multiple studies were available for the same species, we retained the one with the largest number of loci unless the type of site or populations surveyed were distinct, in which case we included all studies.

Species definitions

We sought to avoid defining species by genetic distance or population differentiation in order not to break up species with higher diversity and thereby impose an artificial upper bound on diversity levels. With this in mind, we considered subspecies to be populations of the same species unless reproductive isolation was documented (e.g., *Mus musculus* subspecies [7]). Among species that were genetically defined in the literature and those whose status as species or subspecies has been questioned, we looked for additional evidence for reproductive isolation. In nine cases, we did not find any, and the species are therefore listed together (as “populations”): *Aphanarthrum glabrum glabrum, Aphanarthrum glabrum nudum,* and *Aphanarthrum subglabrum* [8], *Aquila clanga* and *Aquila pomarina* [9], *Canis latrans* and *Canis lupus* [10], *Gorilla beringei* and *Gorilla gorilla* [11], *Hylobates agilis*, *Hylobates lar*, *Hylobates molloch*, and *Hylobates pileatus* [12], *Mesobuthus cyprius* and *Mesobuthus gibbosus* [13], *Nomascus gabriellae* and *Nomascus leucogenys* [14], *Paracoccidioides brasiliensis* PS2, PS3, and S1 [15], and *Pongo abelii* and *Pongo pygmaeus* [16].

Diversity estimates for autosome and sex chromosome comparisons

When comparing autosome and sex chromosome diversity, we preferentially chose estimates for sex chromosome and autosome diversity reported from the same study, in order to use data from the same population samples (e.g., [17]). In some cases, this led us to use a different estimate from that considered in our other analyses. Moreover, for two species (*Ficedula albicolis* and *Ficedula hypoleuca*), a mixed type estimate could be avoided for the autosomes but not for the Z chromosome; to be able to use estimates from the same population, we took the mixed type estimates for both Z and autosomes [18]. If we did not have autosome and sex chromosome diversity estimates from the same study, we included estimates from separate studies in the comparison only if the populations sampled were similar (e.g., for *Oryctolagus cuniculus*). If multiple populations were sampled and reported separately due to population structure, we considered the sex chromosome to autosome ratio for each population.

The final dataset consists of autosomal nucleotide diversity estimates for 167 species, sex chromosome (X or Z) nucleotide diversity estimates for 49 species, and paired sex chromosome and autosome diversity estimates for 29 species.

Additional information about each species

*Mating system*

Species were categorized as obligatory outcrossers, partially selfing, or selfing, based on research papers and botanical literature. Obligatory outcrossers were taken to include species in which separate sexes are required for reproduction and species with a genetic self-incompatibility system. Partial selfers included species that can self-fertilize or reproduce asexually but do not do so exclusively.

*Range*

To categorize species by geographic range, we devised four categories: island, narrow endemic, broad endemic, and cosmopolitan. Some of these are necessarily fuzzy, but our definitions are as follows: Island species live only on one or several islands, with New Guinea considered as the largest island (i.e., not including Australia as an island). Narrow endemics live within a highly limited distribution, roughly defined as less than 2000 km at its widest. Broad endemics live within a range wider than 2000 km, but are not as widespread as cosmopolitan species, which range broadly across three or more continents. Range information was gathered from research papers and online databases. A few species did not have sufficient range information for classification. For consistency, current ranges were considered in all cases, including those for invasive species or species with known, recent habitat degradation.

*Habitat*

Based on the range information and common knowledge, we classified species by whether they live in a terrestrial, marine, or freshwater environment; a few species (3) live in more than one. Internal pathogens were excluded from this categorization.

Diversity and range in *Drosophila*

Within *Drosophila*, we tested for a correlation between diversity and range category (ordered as island, narrow endemic, broad endemic, cosmopolitan) using a generalized least squares method controlling for phylogeny, implemented in R with the package caper (Comparative Analyses of Phylogenetics and Evolution in R; [19,20]). Species relationships were constructed based on refs. [21,22,23] and all branch lengths were set equal to one. We did not perform tests of significance for other analyses because of the lack of phylogenetic independence [24].

Effect of the type of site

To assess whether natural selection on the sites themselves strongly affects the diversity estimates (for example due to the inclusion of non-synonymous sites in mixed estimates or of non-coding sites with regulatory functions), we compared all autosomal estimates to the set of autosomal estimates that were available based on synonymous sites, which are likely to be evolving under weaker and less frequent selection [25,26,27]. The median autosomal diversity levels considering all estimates and only synonymous estimates are shown in Table S1 for the four phyla in which estimates for multiple types of sites were represented and at least three species had synonymous estimates. In Chordata and Magnoliophyta, there is little difference in the median, whichever sites are used. In Arthropoda, the median diversity using only synonymous estimates is slightly higher than the one using all estimates (one-tailed Wilcoxon rank sum test p=0.043), possibly indicating that including non-coding and/or non-synonymous sites results in an underestimate of neutral diversity in this phylum, due to inclusion of sites under direct selection [25]. In Pinophyta, the opposite is found: diversity based on synonymous estimates is lower than for all types of sites (one-tailed p=0.92). Overall, the heterogeneity of site types seems to be influencing diversity estimates by less than two-fold.

Effect of the number of sampling locations

We assessed whether the number of sampling locations might have an effect on the diversity level, namely, if there is systematically higher diversity in species that were sampled in more locations. To test this, we compared the autosomal diversity levels of species with a single population sampled to species with multiple populations sampled, excluding cases where population structure was found (since in this case we considered each population separately). The median autosomal diversity for phyla with at least two species in each category (single population and multiple populations but no detected structure) is shown in Table S2. There are no significant differences between genetic diversity levels in the two sets of species (one-tailed Wilcoxon rank sum test p=0.67 for Arthropoda, p=0.13 for Chordates, and p=0.29 for Pinophyta).

Previous compilations of nucleotide genetic diversity

A few compilations of nuclear genetic diversity estimates based on DNA sequencing have been published previously, the largest in 2006 [28]. They have generally not included many species: 19 eukaryotic species [28] or 32 eukaryotic species [3] or were mostly based on only one or two loci [29,30,31]. As it has become easier to collect such data recently, we were able to include more species, and use estimates based on more loci. In addition, several studies have examined the determinants of mtDNA diversity [32,33]. We do not consider these data as they are for a single locus, which has different dynamics (e.g. a higher mutation rate, no recombination and uniparental inheritance) than the genome as a whole.

**References**

1. Lynch M (2007) The origins of genome architecture. Sunderland, Mass.: Sinauer Associates. xvi, 494 p. p.

2. Neher RA, Shraiman BI (2011) Genetic draft and quasi-neutrality in large facultatively sexual populations. Genetics 188: 975-996.

3. Lynch M, Conery JS (2003) The origins of genome complexity. Science 302: 1401-1404.

4. Tajima F (1983) Evolutionary relationship of DNA sequences in finite populations. Genetics 105: 437-460.

5. Watterson GA (1975) On the number of segregating sites in genetical models without recombination. Theor Popul Biol 7: 256-276.

6. Bachtrog D, Kirkpatrick M, Mank JE, McDaniel SF, Pires JC, et al. (2011) Are all sex chromosomes created equal? Trends Genet 27: 350-357.

7. Forejt J (1996) Hybrid sterility in the mouse. Trends Genet 12: 412-417.

8. Jordal BH, Emerson BC, Hewitt GM (2006) Apparent 'sympatric' speciation in ecologically similar herbivorous beetles facilitated by multiple colonizations of an island. Mol Ecol 15: 2935-2947.

9. Vali Ü, Dombrovski V, Treinys R, Bergmanis U, Daroczi SJ, et al. (2010) Widespread hybridization between the Greater Spotted Eagle Aquila clanga and the Lesser Spotted Eagle Aquila pomarina (Aves: Accipitriformes) in Europe. Biological Journal of the Linnean Society 100: 725-736.

10. Rutledge LY, Garroway CJ, Loveless KM, Patterson BR (2010) Genetic differentiation of eastern wolves in Algonquin Park despite bridging gene flow between coyotes and grey wolves. Heredity 105: 520-531.

11. Thalmann O, Fischer A, Lankester F, Paabo S, Vigilant L (2007) The complex evolutionary history of gorillas: insights from genomic data. Mol Biol Evol 24: 146-158.

12. Brockelman WY, Gittins SP (1984) Natural hybridization in the Hylobates lar species group: implications for speciation in gibbons. In: Preuschoft H, Chivers DJ, Brockelman WY, Creel N, editors. The Lesser Apes: Evolutionary and Behavioural Biology. pp. 498-532.

13. Gantenbein B, Kropf C, Largiader CR, Scholl A (2000) Molecular and morphological evidence for the presence of a new Buthid taxon (Scorpiones: Buthidae) on the Island of Cyprus. Revue Suisse de Zoologie 107: 213-232.

14. Groves CP (1984) A new look at the taxonomy and phylogeny of the gibbons. In: Preuschoft H, Chivers DJ, Brockelman WY, Creel N, editors. The Lesser Apes: Evolutionary and Behavioural Biology. pp. 542-561.

15. Matute DR, McEwen JG, Puccia R, Montes BA, San-Blas G, et al. (2006) Cryptic speciation and recombination in the fungus Paracoccidioides brasiliensis as revealed by gene genealogies. Mol Biol Evol 23: 65-73.

16. Steiper ME (2006) Population history, biogeography, and taxonomy of orangutans (Genus: Pongo) based on a population genetic meta-analysis of multiple loci. J Hum Evol 50: 509-522.

17. Hammer MF, Mendez FL, Cox MP, Woerner AE, Wall JD (2008) Sex-biased evolutionary forces shape genomic patterns of human diversity. PLoS Genet 4: e1000202.

18. Borge T, Webster MT, Andersson G, Saetre GP (2005) Contrasting patterns of polymorphism and divergence on the Z chromosome and autosomes in two Ficedula flycatcher species. Genetics 171: 1861-1873.

19. Freckleton RP, Harvey PH, Pagel M (2002) Phylogenetic analysis and comparative data: a test and review of evidence. Am Nat 160: 712-726.

20. Orme CDL, Freckleton RP, Thomas GH, Petzoldt T, Fritz SA, et al. (in press) caper: Comparative Analyses of Phylogenetics and Evolution in R. Methods in Ecology and Evolution.

21. Markow TA, O'Grady PM (2006) Drosophila : a guide to species identification and use. Amsterdam ; Boston: Elsevier/AP. viii, 259 p. p.

22. Reed LK, Nyboer M, Markow TA (2007) Evolutionary relationships of Drosophila mojavensis geographic host races and their sister species Drosophila arizonae. Mol Ecol 16: 1007-1022.

23. Kopp A, Barmina O (2005) Evolutionary history of the Drosophila bipectinata species complex. Genet Res 85: 23-46.

24. Whitney KD, Boussau B, Baack EJ, Garland T, Jr. Drift and genome complexity revisited. PLoS Genet 7: e1002092.

25. Andolfatto P (2005) Adaptive evolution of non-coding DNA in Drosophila. Nature 437: 1149-1152.

26. Yang Z, Nielsen R (2008) Mutation-selection models of codon substitution and their use to estimate selective strengths on codon usage. Mol Biol Evol 25: 568-579.

27. Zhou T, Gu W, Wilke CO (2010) Detecting positive and purifying selection at synonymous sites in yeast and worm. Mol Biol Evol 27: 1912-1922.

28. Charlesworth B, Charlesworth D (2010) Elements of evolutionary genetics. Greenwood Village, Colo.: Roberts and Co. Publishers. xxvii, 734 p. p.

29. Lynch M (2006) The origins of eukaryotic gene structure. Mol Biol Evol 23: 450-468.

30. Bazin E, Glemin S, Galtier N (2006) Population size does not influence mitochondrial genetic diversity in animals. Science 312: 570-572.

31. Glemin S, Bazin E, Charlesworth D (2006) Impact of mating systems on patterns of sequence polymorphism in flowering plants. Proc Biol Sci 273: 3011-3019.

32. Nabholz B, Mauffrey JF, Bazin E, Galtier N, Glemin S (2008) Determination of mitochondrial genetic diversity in mammals. Genetics 178: 351-361.

33. Nabholz B, Glemin S, Galtier N (2009) The erratic mitochondrial clock: variations of mutation rate, not population size, affect mtDNA diversity across birds and mammals. BMC Evol Biol 9: 54.
